# Fabrication, Characterization, and Properties of Poly (Ethylene-Co-Vinyl Acetate) Composite Thin Films Doped with Piezoelectric Nanofillers

**DOI:** 10.3390/nano9081182

**Published:** 2019-08-20

**Authors:** Giulia Mariotti, Lorenzo Vannozzi

**Affiliations:** The BioRobotics Institute, Scuola Superiore Sant’Anna, 56025 Pontedera (PI), Italy

**Keywords:** nanomaterial, zinc oxide, barium titanate, composite, ethylene vinyl acetate, elastic modulus, toughness, flexural rigidity, radiopacity, piezoelectricity

## Abstract

Ethylene vinyl acetate (EVA) is a copolymer comprehending the semi-crystalline polyethylene and amorphous vinyl acetate phases, which potentially allow the fabrication of tunable materials. This paper aims at describing the fabrication and characterization of nanocomposite thin films made of polyethylene vinyl acetate, at different polymer concentration and vinyl acetate content, doped with piezoelectric nanomaterials, namely zinc oxide and barium titanate. These membranes are prepared by solvent casting, achieving a thickness in the order of 100–200 µm. The nanocomposites are characterized in terms of morphological, mechanical, and chemical properties. Analysis of the nanocomposites shows the nanofillers to be homogeneously dispersed in EVA matrix at different vinyl acetate content. Their influence is also noted in the mechanical behavior of thin films, which elastic modulus ranged from about 2 to 25 MPa, while keeping an elongation break from 600% to 1500% and tensile strength from 2 up to 13 MPa. At the same time, doped nanocomposite materials increase their crystallinity degree than the bare ones. The radiopacity provided by the addition of the dopant agents is proven. Finally, the direct piezoelectricity of nanocomposites membranes is demonstrated, showing higher voltage outputs (up to 2.5 V) for stiffer doped matrices. These results show the potentialities provided by the addition of piezoelectric nanomaterials towards mechanical reinforcement of EVA-based matrices while introducing radiopaque properties and responsiveness to mechanical stimuli.

## 1. Introduction

Recently, nanomaterials are becoming important players in many different technological domains. In fact, nanoelements may enhance the properties of several bulk materials, especially for biomedical applications. The synergy between polymers and inorganic nanofillers can bring to the enhancement of macroscopic properties of neat materials, such as material toughness, thermal resistance, electrical conductivity, optical properties, piezoelectricity, etc. [1]. Specific filler features have been exploited in order to provide bulk materials with responsive and smart properties upon the stimulation offered by an external source for targeted drug delivery purposes [2] as well as for tissue engineering [3,4,5]. For example, our group has already proved the interaction between ultrasound-mediated stimulation and piezoelectric nanocomposite in the field of drug delivery [6].

The inclusion of nanomaterials within polymeric thin films has been identified as a promising strategy for designing functional nanocomposite materials. The thin film technology enables the fabrication of polymeric membranes which thickness can range from few nanometers up to hundreds of micrometers. The relatively high aspect ratio due to the large surface area (few mm or even cm) with respect to the low thickness makes them compliant matrices able to adjust their shape when adhered to a wide range of topographic surfaces. Such combination of multiple properties (e.g. relatively high flexibility, large surface area, high aspect ratio) makes them unique to be used as drug-loaded platforms, actuators, and sensors [7].

The ethylene vinyl acetate (EVA) co-polymer, which is vinyl acetate (VA) block-copolymerized with ethylene, has increased its appealing for possible application within the biomedical field during recent years [8], as for example in controlled release of drugs [9]. EVA has been tested as main component of drug release devices to be used in harsh human departments as the vagina duct [10] and the gastrointestinal tract [11].

Depending on the chased scope, the addition of nanomaterials within the EVA matrix may allow the modification of original matrix properties. However, these properties depend on different features such as size and shape of nanomaterials, EVA polarity, crystallinity, and degree of dispersion of the nanofiller in the polymeric matrix. For example, EVA at different content of VA has been mixed with montmorillonite, which increases the nanocomposite mechanical properties with respect to the bare polymer [12]. In fact, the authors found that both elastic modulus and mechanical strength are a combined function of the clay concentration, proving an increment of the elastic modulus up to five times. Similarly, the addition of halloysites improves the composite mechanical properties, leading to an increase of the Young’s modulus and tensile strength while increasing the nanofiller concentration. Apart from the mechanical behavior, the introduction of clays also enhances both water resistance and permeability to oxygen [13]. Multi-walled carbon nanotubes correspond to another common nanomaterial type widely explored as dopant of polymeric matrices. Such nanofiller can be used to alter both the mechanical behavior and the rheology of EVA in its melt phase. Indeed, EVA can behave as non-Newtonian fluids for higher doping concentrations and as Newtonian fluid for low doping concentrations (up to 0.5% wt.) [14]. Another carbon-based nanomaterial that has been combined with EVA matrices is the graphite [15]. The authors investigated the effect of the nanofiller size and the type of expanded graphite on the thermal behavior and electrical conductivity of EVA nanocomposites. The nanocomposite matrix containing the nanofiller with higher aspect ratio exhibits a stronger strengthening effect due to a higher crystallinity degree. Another type of nanomaterial proposed for doping EVA matrices is the magnetic one. For example, Fe_3_O_4_ nanoparticles can be dispersed in EVA matrices, leading to a reduction of both elongation at break and impact strength, while enhancing the material hardness with respect to neat EVA [16]. The scientific literature is also filled with plenty of examples in which multiple nanofillers have been added in EVA nanocomposites. For example, multiwalled carbon nanotube and montmorillonite are proposed as dopant agents in EVA nanocomposites [17]. Their synergy is demonstrated by resulting mechanical reinforcement of EVA-based matrices.

Despite the great interest in the study of composite materials doped with different type of clays, carbon-based and metallic nanomaterials, the interaction between EVA polymers and ceramic materials has not been widely explored. Only few research studies report possible relations between EVA and piezoelectric nanomaterials. Among them, surface-modified BaTiO_3_ nanoparticles have been only exploited to modify the electrical and thermophysical properties of vulcanized EVA [18], as well as ZnO nanoparticles [19]. To the best of our knowledge, the characterization of both mechanical and piezoelectric properties of EVA-based nanocomposite has not been reported, so far.

This paper reports the fabrication and characterization of nanocomposite EVA thin films doped with piezoelectric nanomaterials. These matrices were evaluated in terms of their morphological, mechanical, thermal, radiopaque, and piezoelectric properties. At this scope, a series of EVA copolymers has been investigated (18%, 25%, and 40% of VA), varying their concentration (5% and 10% wt. in toluene) and doping them with two types of piezoelectric nanomaterials, namely barium titanate (BaTiO_3_) nanoparticles and zinc oxide (ZnO) nanopowder, at different concentrations (10% and 20% wt.).

## 2. Materials and Methods

### 2.1. Materials

Polyethylene vinyl acetate (EVA, vinyl acetate content: 18%, 25%, and 40%) and ZnO nanopowder (diameter less than 100 nm) were purchased from Sigma-Aldrich (Merck KGaA, Darmstadt, Germany). BaTiO_3_ nanoparticles (nominal diameter: 300 nm, purity > 99.9%) were purchased from Nanostructured & Amorphous Materials (Houston, TX, USA). Nanomaterials were imaged through a Dual Beam microscopy workstation (Figure S1).

### 2.2. Thin Film Fabrication

The EVA copolymers (18%, 25%, and 40% of VA, named EVA18, EVA25, and EVA40) were dissolved in toluene (5% and 10% wt., namely 5EVA and 10EVA) by heating the hot plate up to 60 °C to favor the material dissolution while magnetically stirring the solution. Then, in case of nanocomposite materials, ZnO or BaTiO_3_ were added to the polymer solution (10% and 20% wt. with respect to the polymer content) and sonicated with an ultrasonic bath for 1 h while keeping the temperature higher than 40 °C. Nanocomposite membranes were prepared by casting. Each solution (3 mL) was casted on a glass Petri dish (diameter: 60 mm) and let to evaporate for 24 h within a chemical hood.

### 2.3. Thickness and Morphological Evaluation

The thickness was measured by means of a profilometer (KLA Tencor, Milpitas, CA, USA). For each sample type, five independent samples were tested.

The top film surface was imaged by means of a scanning electron microscopy (SEM, EVO MA 15, Carl Zeiss, Oberkochen, Germany). SEM scans were carried out by setting a beam voltage of 10 kV and a current of 30 pA. This allowed investigating the material surface, for identifying the nanomaterial dispersion within the polymer matrix. The acquired images were analyzed in order to calculate the interparticle distance (*l*) in each composite formulation, according to the following equation [20]:

(1) l=d[(π6V)13−1],

where *V* and *d* are the volume fraction and diameter of the nanofiller.

### 2.4. Mechanical Characterization

Mechanical testing was performed on nanocomposite EVA membranes by means of a traction machine (Instron 2444, load cell ± 1 kN): Material Young’s modulus, elongation at break, ultimate strength and toughness were assessed for each sample type. The specimens were stretched at a constant load speed of 10 mm/min following ASTM D638. The elastic modulus was calculated by analyzing the first linear region of the stress-strain curves (deformation up to 10%), according as follows:

(2) $$\sigma =\frac{F}{hb},$$

and

(3) $$\varepsilon =\frac{L-L_{0}}{L_{0}},$$

where *σ*, *F*, *h*, *b*, *ε*, *L*, and *L*_0_ are the stress, tensile load, thickness, width, tensile strain, length when stressed and initial length, respectively. The test was repeated on eight different samples for each material formulation.

Flexural rigidity (D) was expressed as:

(4) $$D=\frac{Eh^{3}}{12\left(1-v^{2}\right)},$$

where *E* and *ν* are the Young’s modulus and Poisson’s ratio (defined as 0.35, [21]), respectively.

### 2.5. Differential Scanning Calorimetry

The thermal behavior (melting point and crystallinity) of the selected 10EVA formulations was investigated by Differential Scanning Calorimetry (DSC). The thermograms were recorded on a Mettler Toledo DSC1 Star System instrument (Greifensee, Switzerland). For each measurement, ~10 mg of material was placed in a standard aluminum sealed capsule and underwent a specific thermal cycle, described as follows: (1) Heating from −30 to 180 °C at a heating rate of 10 °C/min; (2) cooling to −30 °C at a cooling rate of 10 °C/min; (3) heating from −30 to 180 °C at a heating rate of 10 °C/min. The material melting point was obtained from the second heating run, while crystallinity was calculated with reference to the enthalpy of fusion of the perfect polyethylene crystal (277.1 J/g, [11]). The test was repeated on three different samples for each material formulation.

### 2.6. Radiopacity Measurement

For the radiopacity test, the exposure parameters were set up at 50–60 kV (tube voltage), 100 mA, and 0.063 s. The wavelength was set to 2.5 × 10^−11^ m. The object to focus distance was 15 cm. The radiographs were processed, and a digital image of the radiograph was obtained. The signal-to-noise ratio of the tested materials were analyzed using specific imageJ software (ImageJ version 1.51i, National institutes of Health, Bethesda, MD, USA). Numbers between 0 (pure black) and 255 (pure white) were assigned accordingly, and the signal-to-noise ratio was calculated by subdividing the grey value for the background. In this experiment, only 10EVA40 samples with the highest content of ZnO and BaTiO_3_ were analyzed to verify the radiopacity introduced by the nanofiller type.

### 2.7. Electromechanical Response Analysis

Two electrodes made of a bilayer of Ti (~10 nm) and Au (~100 nm) were deposited in the opposite planar faces of 10EVA thin films through sputtering. A pressure of 400 kPa has been applied formulation using a cylinder with a diameter of 4 mm to test the electromechanical response of the thin film, connected to a linear rail. To verify the piezoelectric behavior of the nanocomposite membranes, signals were also acquired by inverting the poles of acquisition (Figure S2). The electromechanical response of each nanocomposite EVA matrices was analyzed by using a custom-made circuit, and signals were processed in Matlab (2018a).

### 2.8. Statistical Analysis

Normal data were reported as average value ± standard deviation. Data were analyzed through a one-way ANOVA with Tukey’s post-test (GraphPad Prism v6). Statistically significant differences among sample types were defined through a significance threshold set at 5% (** = *p* < 0.01, * = *p* < 0.05).

## 3. Results and Discussion

### 3.1. Thin Film Fabrication: Analysis of Thickness and Nanomaterial Dispersion

**Figure 1:**
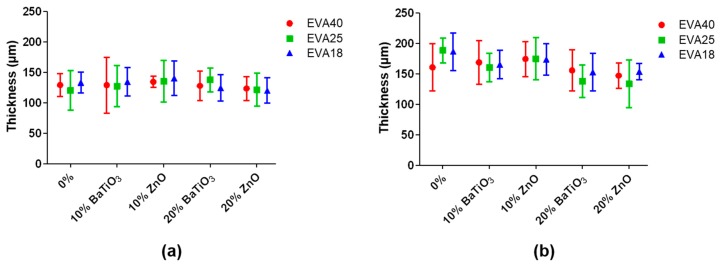
Thickness of the nanocomposite ethylene vinyl acetate (EVA) thin film for the EVA concentration of 5% wt. (**a**) and 10% wt. (**b**).

Composite EVA thin films were successfully fabricated by film casting in a Petri dish with specific dimensions and controlling the volume of deposition. The results in terms of thickness are summarized in Figure 1.

The achieved thickness ranges from about 100 to 200 µm, without significant differences between the different formulations. Despite testing of EVA solutions at different polymer concentration (5% and 10% wt.), the obtained thicknesses are not statistically different. This demonstrates that the polymer concentration in toluene is not an effective factor for significantly differing the thickness among all the nanocomposite formulations tested. Furthermore, the film casting procedure allowed the fabrication of thin films with a relatively flat surface. The rather high polymer concentrations and the low boiling point of toluene do not allow the formation of macropores during solvent evaporation, which are not present on the surface. 

**Figure 2:**
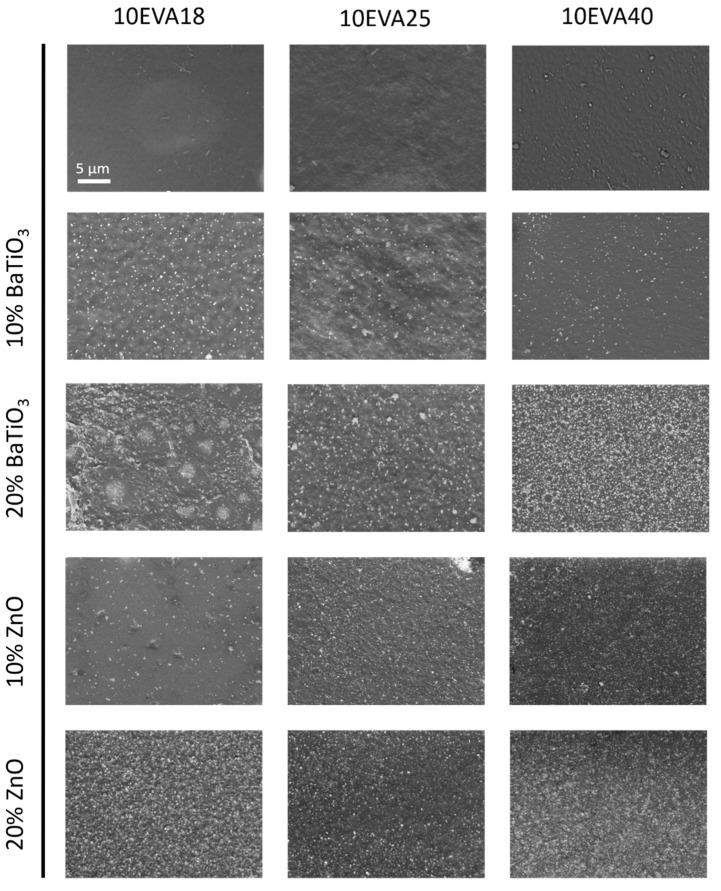
Scanning electron microscopy (SEM) images of each 10EVA formulation.

After the fabrication step, SEM images were acquired to qualitatively investigate the dispersion of nanomaterials within the polymeric matrices. A representation of the surface of all 10EVA formulations is reported in Figure 2.

Both nanomaterials (BaTiO_3_ and ZnO) result homogeneously dispersed in each EVA formulation without any evident sign of aggregation. In addition, the evaporation of the solvent did not cause any formation of macropores on the material surface, preserving a relatively flat topography. A similar trend was found for all the nanocomposite formulations at 5% of EVA (data not shown). The high shear forces introduced by ultrasonic energy during the nanocomposite polymeric solution were enough to uniformly and stably separate nanomaterials in the solution. Furthermore, a good interfacial interaction between the nanomaterial and the polymer allowed the good dispersion of both dopant agents. From the images, it can be noted the higher number of ZnO elements, approximately three times more the amount of BaTiO_3_ particles, despite the two nanomaterials were equally weighted during the phase of preparation. The mentioned difference in terms of number of particles is mainly caused by the smaller diameter of ZnO nanopowder (nominally about 1/3 of the diameter of BaTiO_3_ nanoparticles) because these materials have similar densities (5.61 g/cm^3^ for ZnO and 6.02 g/cm^3^ for BaTiO_3_). This was also demonstrated by the estimation of the interparticle distance (Equation (1)), that is 217 nm (10% wt.) and 153 nm (20% wt.) for the ZnO, and 675 nm (10% wt.) and 478 nm (20% wt.) for BaTiO_3_, respectively.

### 3.2. Mechanical Properties

**Figure 3:**
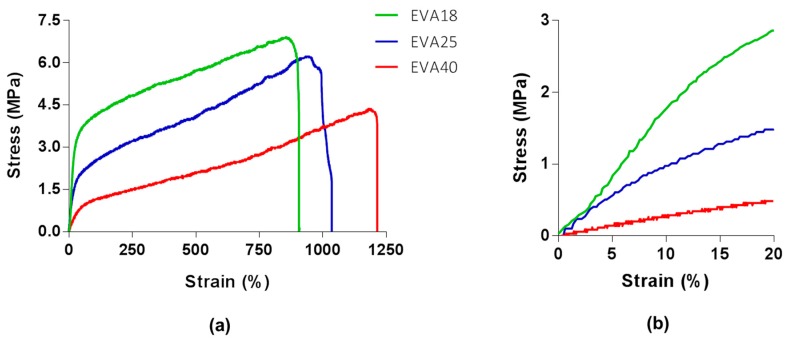
(**a**) Representative stress-strain curves of ethylene vinyl acetate (EVA) formulations at different vinyl acetate (VA) contents, and (**b**) a magnification of the first 20% of deformation.

Each nanocomposite material formulation underwent traction tests. As already familiar from the scientific literature [22], EVA mechanical properties are influenced by the weight percent of VA. Figure 3a shows representative stress-strain curves of EVA18, EVA25, and EVA40 derived from Equations (2) and (3), with a magnification on the first 20% of deformation (Figure 3b).

**Figure 4:**
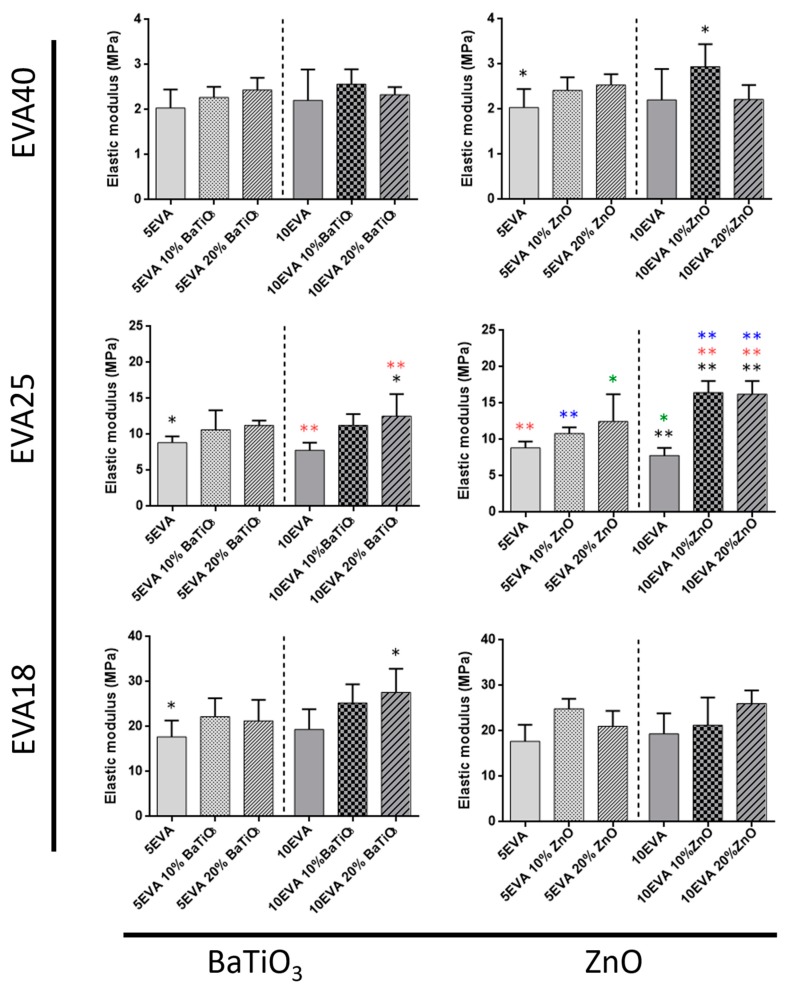
Elastic modulus of nanocomposite EVA-based matrices subdivided for the content of vinyl acetate and the ceramic nanomaterial included. * = *p* < 0.05, ** = *p* < 0.01. Different colors have been used to evidence the statistical significance between the compared cases in each graph.

**Figure 5:**
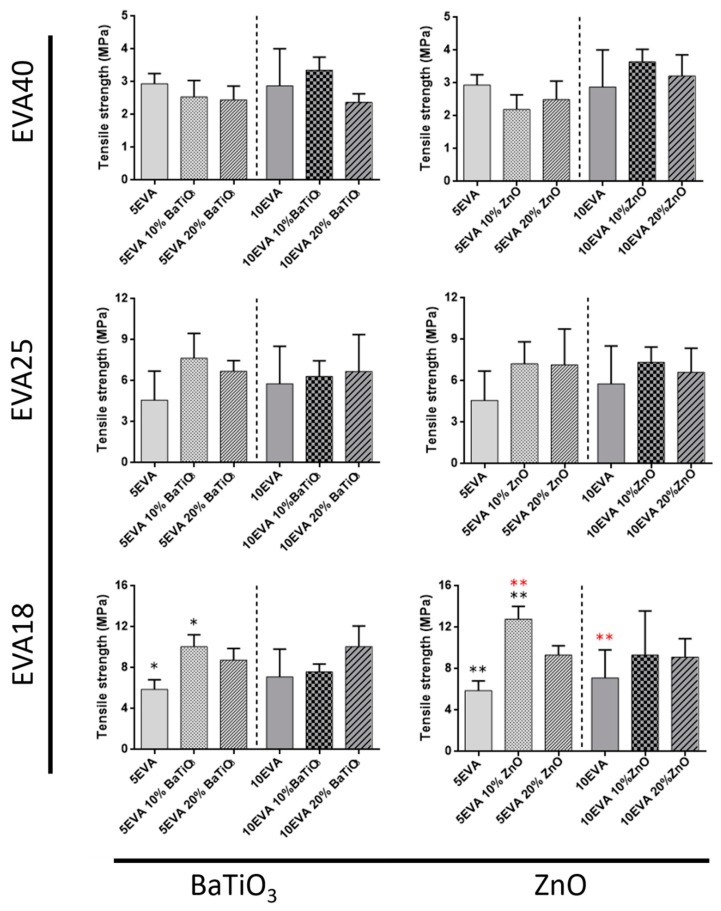
Tensile strength of nanocomposite EVA-based matrices subdivided for the content of vinyl acetate and the ceramic nanomaterial included. * = *p* < 0.05, ** = *p* < 0.01. Different colors have been used to evidence the statistical significance between the compared cases in each graph.

**Figure 6:**
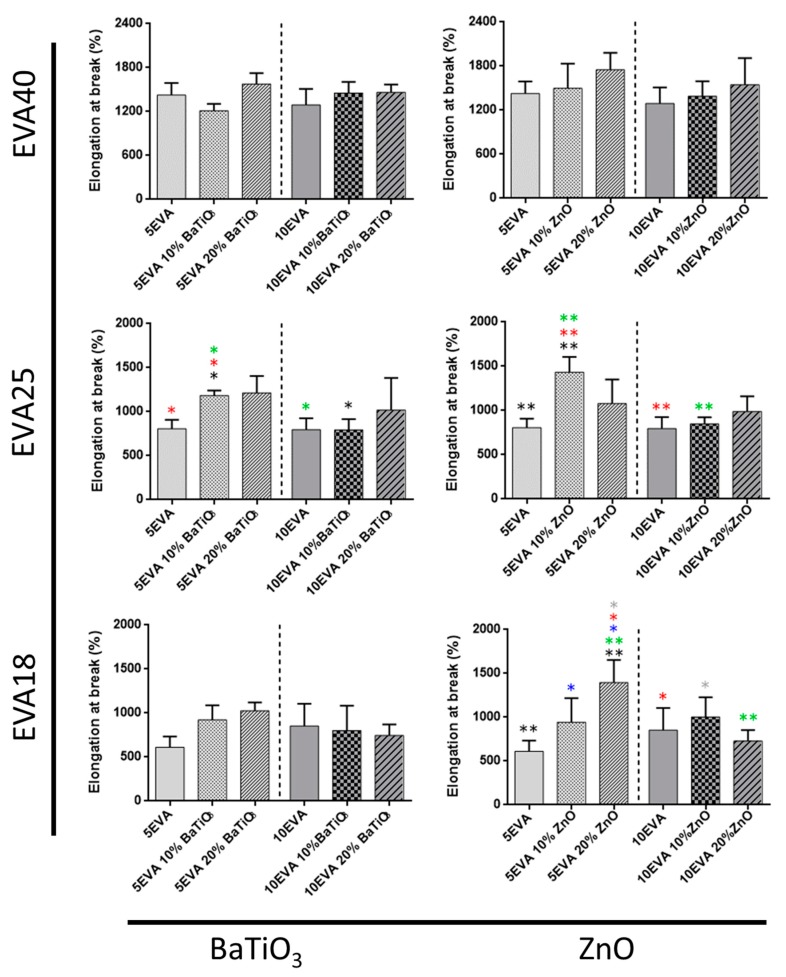
Elongation at break of nanocomposite EVA-based matrices subdivided for the content of vinyl acetate and the ceramic nanomaterial included. * = *p* < 0.05, ** = *p* < 0.01. Different colors have been used to evidence the statistical significance between the compared cases in each graph.

Tensile tests allowed getting insights on many mechanical parameters of nanocomposite materials. The elastic modulus (Figure 4), the elongation at break (Figure 5), and the tensile strength (Figure 6) were analyzed, and data are summarized in Table 1.

The analysis of the mechanical properties provides interesting understandings on the mechanical behavior of nanocomposite EVA matrices. As shown in Figure 4, the stiffness of the nanocomposite polymer mainly increases by decreasing the content of vinyl acetate within the EVA polymer, thus the polarity of the matrix (from EVA40 to EVA18). Instead, the variation of the polymer concentration (5% and 10% wt. in toluene) does not involve a significant change in the bare material stiffness. A similar result was also reported by Faker et al., who analyzed the mechanical behavior of EVA18 dependently on polymer concentration [23].

On the one hand, the introduction of nanomaterials altered the mechanical properties of such nanocomposite matrices, showing in some cases an effective interaction between the polymer and the ceramic nanofiller. Data show a minimal influence due to the addition of nanomaterials while using EVA18 and EVA40 as polymeric matrix. On the other hand, the matrices based on EVA25 are strongly influenced by both nanofillers, leading to significant changes in the elastic modulus up to two times (10EVA25 vs. 10EVA25 10% and 20% ZnO). Generally, material mechanical properties increase in each material formulation. 

The same materials were also analyzed in terms of tensile strength, and a summary of the results is reported in Figure 5.

The tensile strength of nanocomposite polymers generally increases with the polarity of the matrix, without any significant effect due to the introduction of nanomaterials. In this case, the only exception corresponds to the case of EVA18, in which the addition of 10% ZnO strongly impacted on the 5EVA18 formulation. On the other hand, the tensile strength slightly decreases in the EVA40 compositions, especially when the 5EVA polymer was tested.

Finally, the elongation at break of all the EVA formulations is reported in Figure 6.

The elongation at break of the nanocomposites generally decreases with the polarity of the matrix. The addition of nanomaterials positively contributed to improve such EVA feature. Indeed, the chain mobility of the polymeric macromolecules is affected by the incorporation of both nanomaterials, leading to a general maintenance of elongation rates. The addition of ZnO in polymeric matrices has been demonstrated to positively influence the elongation at break of the composite materials [24]. Here, the only exception is represented by the softer formulation (EVA40), for which this effect was not significantly visible.

Finally, the stress-strain curves allowed also to get further insights on the toughness of all the material formulations (Table 1). The toughness is generally higher for material formulations made of EVA18 and EVA25, and this feature is further improved by the addition of the nanofiller (up to 100 MPa) probably because of the achievement of a homogenous dispersion and good adhesion between matrix and nanoparticles. The differences are more highlighted while testing the polymer content of 5% (5EVA). For EVA40-based matrices ones, values are not statistically relevant in all cases.

The last analyzed parameter is the flexural rigidity (Equation (4)). This is dependent on the VA content, thus the stiffness, even if the predominant role is assumed by the thickness. For such reason, the flexural rigidity of 10EVA-based thin films result higher than 5EVA ones due to the formation of thicker films. Results are summarized in Table 1.

Generally, an increase of VA concentration (from EVA18 to EVA40) results in decreased stiffness and tensile strength, but an increased elongation at break. The toughness is generally similar between EVA18 and EVA25, while decreasing for the softer formulations (EVA40). Such mechanical properties can be furtherly tuned by adding ceramic nanomaterials. The introduction of nanofillers usually might provide additional and/or peculiar features to the polymeric matrix that are not usual for the polymer, such as mechanical, optical, and piezoelectric ones [25]. In the field of biomaterials, the mechanical reinforcement of polymers due to the use of inorganic nanofillers is of great interest for many applications. The peculiar features of nanomaterials, as a large surface to volume ratio combined with their intrinsic rigidity, enable multiple particle–matrix interactions when dispersed into the polymeric matrix, thus leading to an overall improvement of material properties [26,27,28]. There are many mechanisms with which nanomaterials can improve the mechanical strength of polymer, as transferring the stress from the matrix to the stiffer filler, thus substituting the softer polymeric components of the polymeric matrix. Indeed, nanomaterials can help in absorbing the energy due to the applied stress, enabling its dispersion in a larger volume of the nanocomposite matrix, thus increasing the material toughness. In previous studies, it has been shown that the Young’s modulus, mechanical strength and ductility of barium titanate-doped EVA matrices (40% VA content) increase with increasing BaTiO_3_ content up to loading levels of 20% vol., while testing loading level of 30% vol., both the mechanical strength and ductility of the nanocomposites decrease in relation with the loading [18]. Another explanation is provided by the concept of interphase. The interphase is a third phase with different properties respect with polymer matrix and nanoparticle phases. The interphase may be formed due to high interfacial areas and strong interfacial interactions between polymer and nanoparticles and may play an important role in their properties. For example, small nanoparticles and large interphase thickness have positive effects on Young’s modulus of nanocomposite polymers [29]. 

In some other cases, mechanical reinforcing of nanocomposite polymers can be provoked by the aggregation of nanofillers, rather than the interfacial adhesion between polymer and nanoparticles [30]. On the other hand, the fabrication of nanocomposite materials may lead to aggregation/agglomeration phenomena of the included nanofiller that can generate defects and stress concentrations, which may sometimes decrease the mechanical properties of composite materials [31]. Those effects are generally increased by increasing the nanofiller content and reducing the filler size. In addition, nanofiller morphology has a very important role in the overall mechanical behavior of nanocomposite materials. 

In the proposed nanocomposite thin films, the choice of the components and the preparation procedure allow the fabrication of membranes in which the addition of nanomaterials provoked beneficial effects, in terms of stiffness, elongation at break, and tensile strength. Furthermore, the toughness is significantly improved in the case of EVA18 and EVA25.

A further exploration of the mechanical behavior of nanocomposite EVA thin films was carried out by analyzing the flexural rigidity. The flexural rigidity describes the resistance to bend thin films. Here, the effect was mainly provided by the VA content of each matrix, which led to a significant change in the material stiffness, thus a consequent variation in the flexural rigidity. Indeed, the effect of the thickness, which is relevant for the estimation of the flexural rigidity, was not extremely relevant due to the not significant changes among all the EVA formulations. The analysis of the flexural rigidity has already been correlated with the polymer concentration in thin films, as shown by Hasebe et al. [32]. In such case, the fabrication of thin films with different polymer concentration led to a significant variation of the substrate thickness, which derived the difference in the estimated flexural rigidity.

The adoption of EVA matrices alternatively to the most standard polydimethylsiloxane (PDMS) may involve several advantages for the fabrication of piezoelectric nanocomposite materials. PDMS is mechanically tunable depending on the ratio monomer/curing agent ratio (Young’s modulus can range from tens of kPa to MPa); on the other hand, EVA has a decisively higher elongation at break than PDMS (up to 100%). Above all, EVA is also more economic than PDMS, thus representing a cheaper alternative for building nanocomposite materials.

### 3.3. Thermal Properties

**Figure 7:**
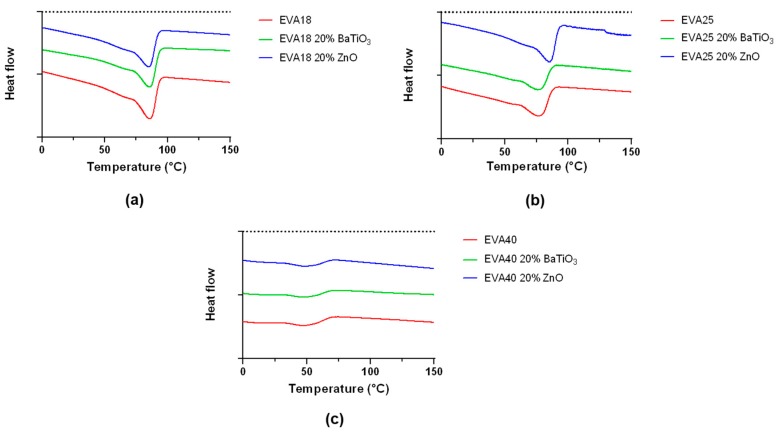
Comparison of the thermograms of ethylene vinyl acetate (EVA) formulations based on: (**a**) EVA18, (**b**) EVA25, (**c**) EVA40.

DSC is used to measure the melting point and the crystallinity of nanocomposite EVA polymers. Analyses were performed on 10EVA matrices, focusing on the maximum content of the included nanofiller. An example of the trend of distinct thermograms is reported in Figure 7.

Thermal investigation shows a clear difference between the EVA polymers at different VA content. In general, EVA exhibits multiple melting endotherms, composed of a slight endotherm started at a lower temperature and a major melting peak at the end and a broad medium peak which overlaps the others. A summary of the results is reported in Table 2.

For the neat polymer, the melting point ranges from 46.7 °C (EVA40) to 83.1 °C (EVA18), while the crystallinity ranges from 9.8% (EVA40) to 23.4% (EVA18). As the content of VA increases, the melting point of the EVA copolymer decreases, because the polyethylene crystallinity is disrupted by the VA component. These values are in accordance with results already showed in the scientific literature [11]. In fact, the incorporation of VA units into the polyethylene backbone chain has the effect to reduce both crystallinity and melting point while increasing the material flexibility, as previously showed by the elastic modulus/elongation at break results (Figure 4 and Figure 6). Interestingly, the introduction of ceramic nanofillers slightly increases both the melting point and the crystallinity of the EVA polymer, with a more relevant effect at smaller content of VA (EVA18 and EVA25). To be more specific, the statistical difference for both melting point and crystallinity between EVA25 and EVA25 with the 20% wt. of ZnO reflects the statistical significance found for the elastic modulus in Figure 2, as well as the statistical difference found for the crystallinity between EVA18 and EVA18 with the 20% wt. of BaTiO_3_. It was observed that the crystallization mechanism of nanocomposite polymers may strongly depend on the intrinsic features of the nanofiller and in consequence its dispersion in the polymeric matrix [33]. For example, in well-dispersed nanocomposites the growing lamellae can influence the disposition of the included nanoparticles, thereby broadening interstitials to allow bulk-like lamellae to form [34]. These results may also suggest that nanomaterials can act as nucleation site. In fact, nucleation of crystallization can appear with the inclusion of inorganic nanomaterials, widening the usual confined crystallization offered by the neat polymer [35]. This analysis allows to better clarify the important role of piezoelectric nanofillers for varying bulk properties of composite polymeric matrices. Generally, EVA consists of two phases [36]: An interfacial and more rigid phase, and a very mobile amorphous phase. The introduction of nanofillers may alter the mobility of the amorphous phase between crystalline chains, thus leading to a general increase of the degree of crystallinity of doped formulations. For example, polymer chains close to nanofillers can be stretched and can decrease the conformational entropy of chains. The presence of a rigid interface due to the ceramic origin of nanofiller could drive the segregation of lower molecular weight chains during the thin film formation upon solvent evaporation [27].

### 3.4. Radiopacity

**Figure 8:**
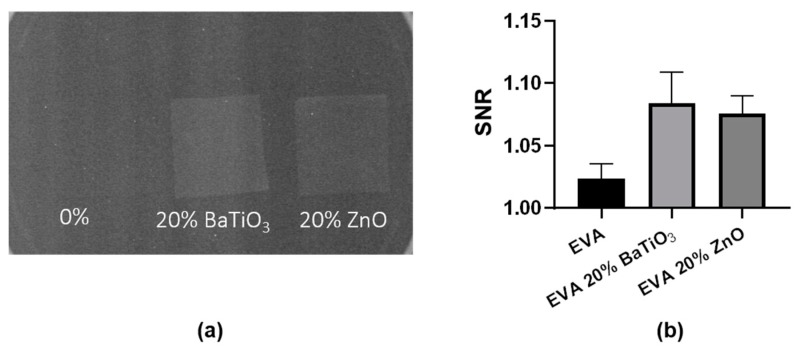
(**a**) Digital image of an X-ray analysis of the EVA-based thin films, and (**b**) analysis of their radiopacity.

Polymeric composites can be made radiopaque by the incorporation of piezoelectric nanofillers possessing high atomic numbers such as zinc and barium [37]. The evaluation of radiopacity is shown in Figure 8.

According to our results, the addition of both ceramic nanofillers confers radiopacity to the nanocomposite material, increasing the signal-to-noise ratio in comparison with the neat EVA. This result demonstrates that nanocomposite EVA materials can be used to manufacture implantable devices that are radiopaque, making possible their visualization using radiography. Many biomedical devices lack radiopacity, making the visualization and assessment of material within the human body difficult. This may lead to a difficult evaluation of the nanocomposite material fate without using invasive methods [38].

### 3.5. Electromechanical Response

**Figure 9:**
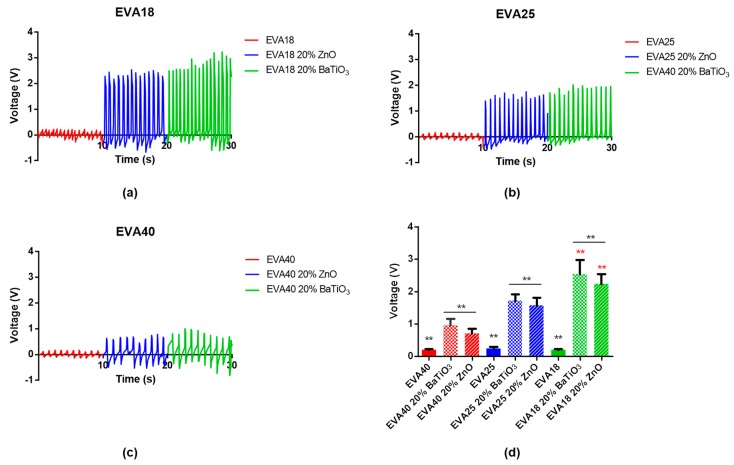
Electromechanical response of nanocomposite ethylene vinyl acetate (EVA) matrices: (**a**) EVA18, (**b**) EVA25, and (**c**) EVA40. (**d**) Summary of the output voltage (** = *p* < 0.01).

The electromechanical response of nanocomposite 10EVA matrices was evaluated by applying a pressure on top of the thin film and recording the signal generated by the presence of piezoelectric nanomaterials within the matrix. When the external force is applied to the thin film, each piezoelectric nanomaterial undergoes deformation, generating a net local polarization on it, thus a potential difference. This leads to a piezoelectric voltage that can be detected by electrodes. Results are reported in Figure 9.

In general, the effect of piezoelectric dopant agents on the electromechanical response of nanocomposite EVA matrices is evident. In fact, in absence of nanofiller the output voltages do not overcome 0.2–0.25 V. On the other hand, their presence within the polymeric matrix allows the achievement of increasing output voltages in relation to the material stiffness, ranging from almost 0.71 ± 0.14 V (EVA40 20% ZnO) to 2.55 ± 0.43 V (EVA18 20% BaTiO_3_). In fact, the highest stiffness of EVA18 allows the transmission of higher stresses to piezoelectric elements that can consequently produce a higher voltage output. Interestingly, the difference in using a different type of dopant is only statistically evident when fabricating stiffer matrices, while in the other cases there are no evident differences. 

To the best of our knowledge, there are no reports which aim at demonstrating the electromechanical properties of doped EVA matrices. For example, it is only shown that the increased loading of BaTiO_3_ nanoparticles (diameter: 100 nm) can improve the conductivity and permittivity of EVA thin films [18]. Our analysis demonstrates how EVA substrates with different content of VA and doping may vary the responsivity to mechanical stresses, with results comparable to those found in the scientific literature for some PDMS-doped matrices [39]. 

The use of nanocomposite matrices based on EVA may have a wider applicability than Polyvinylidene Fluoride (PVDF) for certain applications. PVDF is a FDA-approved thermoplastic polymer which presents interesting piezoelectric properties, widely investigated for biomedical application [40]. Despite this, PVDF results are decisively stiffer (Young’s modulus in the order of GPa) and possess a lower elongation at break (from 25% to 500%). Such features make the PVDF less appropriate in applications in which a certain degree of flexibility, thus a low flexural rigidity value, is required (e.g., sensing in tissues with irregular shapes [41]).

These nanocomposite membranes may find space in many biomedical applications, being EVA FDA-approved. For example, such nanocomposite membranes may find potential applications in self-powered touch sensors [42] or energy harvesters [43]. The biocompatibility and flexibility of EVA makes it a suitable candidate as material to be implanted inside the body for healthy monitoring [44]. Another interesting domain of application is the soft robotics. Soft robots require soft sensors that can be embedded into the robot body without adding rigidity and kinematic limitations [45]. Alternatively, such nanocomposite membranes may be further explored for possible applications in tissue engineering. Indeed, the piezoelectricity induced by the addition of piezoelectric nanofillers could be exploited to generate local electrical charges upon external mechanical stimuli (e.g., ultrasound waves), enabling regenerative phenomena which can help the restoration of functions in piezoelectric tissues, as for example the articular cartilage one [46]. 

The performance of these nanomembranes could be furtherly improved by acting on the inclusion of material with higher piezoelectric coefficient or increasing the film thicknesses. In fact, the relatively low thickness of EVA matrices (up to 200 µm) could not lead to high output voltages with respect to other nanocomposite matrices with higher thickness (up to 1 mm, [47]). In fact, the voltage output of is a function of its capacitance, as the piezoelectric layer is very thin, there could be high capacitance and low charge. In case of EVA thin films, since the voltage output is linearly correlated with the nanocomposite thickness, an increase of material thickness will lead to increased output for the same applied pressure. Furthermore, EVA matrices cannot be subjected to poling, which can increase the piezoelectric properties of nanomaterials as BaTiO_3_ of a factor of at least 10 [48]. The use of different nanomaterials, as well as different shapes/sizes may allow the achievement of higher output voltages. 

## 4. Conclusions

Nanocomposite thin films of EVA at different VA content, doped with different loading of BaTiO_3_ and ZnO, were prepared by solvent casting. These nanocomposite matrices were characterized in terms of morphological, mechanical, thermal, radiopaque, and piezoelectric properties. The tuning of material formulation highlights the possibility to vary thin film mechanical properties, crystallinity, and melting point. The doped EVA composites were also radiopaque, enabling their visualization under x-ray. The electromechanical response induced by the presence of piezoelectric nanomaterials has been verified, demonstrating the achievement of output voltages up to 2.55 V for the doped 18EVA substrates with barium titanate (20% wt.). 

The use of piezoelectric nanomaterials as dopant agent in EVA matrices is still rather poorly explored. The combination of EVA with ceramic nanomaterials as piezoelectric nanoparticles may open future scenarios for possible applications in sensing and monitoring as well as drug release systems or engineering of human tissue, being a FDA-approved material.

## Figures and Tables

**Table 1:** Mechanical analysis on the different material formulations: elastic modulus, tensile strength, elongation at break, toughness, and flexural rigidity.

Polymer Formulation	Elastic Modulus (MPa)	Tensile Strength (MPa)	Elongation at Break (%)	Toughness (MPa)	Flexural Rigidity (MPa)
5EVA18	20.85 ± 5.51	5.89 ± 0.94	611 ± 121	34.44 ± 7.36	4.00 × 10^−6^
5EVA18 10% BaTiO_3_	24.11 ± 1.91	10.05 ± 1.18	922 ± 167	55.54 ± 6.14	5.18 × 10^−6^
5EVA18 20% BaTiO_3_	21.29 ± 4.70	8.75 ± 1.13	1026 ± 95	65.61 ± 16.07	6.59 × 10^−6^
5EVA18 10% ZnO	24.86 ± 2.19	12.80 ± 1.24	944 ± 274	79.28 ± 13.48	3.92 × 10^−6^
5EVA18 20% ZnO	22.12 ± 3.18	9.33 ± 0.90	1396 ± 256	100.33 ± 15.14	3.48 × 10^−6^
10EVA18	20.44 ± 4.84	7.12 ± 2.71	852 ± 254	54.92 ± 37.51	1.20 × 10^−5^
10EVA18 10% BaTiO_3_	25.24 ± 4.18	7.59 ± 0.77	801 ± 281	53.14 ± 27.17	1.09 × 10^−5^
10EVA18 20% BaTiO_3_	26.32 ± 5.69	10.07 ± 2.01	745 ± 125	56.46 ± 14.22	1.07 × 10^−5^
10EVA18 10% ZnO	22.94 ± 6.28	9.33 ± 4.27	1002 ± 227	67.72 ± 26.35	9.4 × 10^−6^
10EVA18 20% ZnO	24.87 ± 3.81	9.13 ± 1.77	728 ± 124	55.69 ± 17.28	9.04 × 10^−6^
5EVA25	8.85 ± 0.86	4.58 ± 2.13	806 ± 103	42.59 ± 21.74	1.46 × 10^−6^
5EVA25 10% BaTiO_3_	10.63 ± 2.74	7.66 ± 1.81	1184 ± 57	83.91 ± 9.80	2.09 × 10^−6^
5EVA25 20% BaTiO_3_	11.25 ± 0.67	6.70 ± 0.78	1215 ± 190	73.05 ± 10.26	2.56 × 10^−6^
5EVA25 10%ZnO	10.83 ± 0.82	7.23 ± 1.59	1432 ± 173	90.37 ± 26.86	2.80 × 10^−6^
5EVA25 20%ZnO	12.47 ± 3.76	7.16 ± 2.59	1078 ± 272	77.78 ± 37.94	2.14 × 10^−6^
10EVA25	7.81 ± 1.06	5.79 ± 2.74	797 ± 128	58.35 ± 25.36	4.98 × 10^−6^
10EVA25 10% BaTiO_3_	11.25 ± 1.58	6.31 ± 1.16	794 ± 122	50.64 ± 17.26	4.44 × 10^−6^
10EVA25 20% BaTiO_3_	12.54 ± 3.10	6.68 ± 2.70	1019 ± 362	66.47 ± 23.16	8.40 × 10^−6^
10EVA25 10%ZnO	16.45 ± 1.58	7.35 ± 1.10	847 ± 75	52.35 ± 11.74	3.14 × 10^−6^
10EVA25 20%ZnO	16.24 ± 1.81	6.62 ± 1.75	989 ± 172	66.87 ± 13.70	3.70 × 10^−6^
5EVA40	2.03 ± 0.41	2.94 ± 0.31	1426 ± 164	39.82 ± 13.84	4.18 × 10^−7^
5EVA40 10% BaTiO_3_	2.27 ± 0.24	2.54 ± 0.50	1212 ± 95	22.19 ± 2.13	4.63 × 10^−7^
5EVA40 20% BaTiO_3_	2.44 ± 0.27	2.45 ± 0.43	1577 ± 147	30.45 ± 4.02	5.60 × 10^−7^
5EVA40 10% ZnO	2.42 ± 0.29	2.19 ± 0.45	1498 ± 334	28.74 ± 5.77	4.86 × 10^−7^
5EVA40 20% ZnO	2.64 ± 0.05	2.49 ± 0.57	1749 ± 231	38.85 ± 14.84	4.55 × 10^−7^
10EVA40	2.20 ± 0.68	2.88 ± 1.13	1292 ± 218	34.73 ± 18.49	8.74 × 10^−7^
10EVA40 10% BaTiO_3_	2.56 ± 0.33	3.35 ± 0.40	1452 ± 155	31.45 ± 5.67	1.17 × 10^−6^
10EVA40 20% BaTiO_3_	2.30 ± 0.17	2.37 ± 0.27	1463 ± 108	24.65 ± 1.19	1.49 × 10^−6^
10EVA40 10% ZnO	2.78 ± 0.56	3.64 ± 0.38	1389 ± 204	33.44 ± 2.06	8.78 × 10^−7^
10EVA40 20% ZnO	2.22 ± 0.32	3.21 ± 0.64	1546 ± 363	38.91 ± 13.07	6.72 × 10^−7^

**Table 2:** Differential Scanning Calorimetry (DSC) analysis on the different material formulations. Statistical differences are defined by *p*-value > 0.05 (*) in each EVA subgroup.

Polymer Formulation	Melting Point (°C)	Crystallinity (%)
EVA18	83.1 ± 2.2	23.4 ± 4.1 (*)
EVA18 20% BaTiO_3_	85.1 ± 0.3	33.8 ± 1.9 (*)
EVA18 20% ZnO	85.5 ± 0.5	29.7 ± 4.5
EVA25	77.3 ± 1.4 (*)	24.8 ± 0.4 (*)
EVA25 20% BaTiO_3_	78.9 ± 5.3	28.9 ± 5.7
EVA25 20%ZnO	85.2 ± 0.7 (*)	31.7 ± 0.8 (*)
EVA40	46.7 ± 0.3	9.8 ± 0.5
EVA40 20% BaTiO_3_	46.7 ± 0.8	10.2 ± 0.4
EVA40 20% ZnO	46.1 ± 0.9	8.8 ± 3.2

## Supplementary Materials

The following are available online at https://www.mdpi.com/2079-4991/9/8/1182/s1, Figure S1: Dual Beam imaging of zinc oxide nanopowder (left) and barium titanate nanoparticles (right), Figure S2: Voltage output in standard (left) and inverted (right) poles configuration.
