# Supplementary material for: Fabrication, Characterization, and Properties of Poly (Ethylene-Co-Vinyl Acetate) Composite Thin Films Doped with Piezoelectric Nanofillers
Source: Nanomaterials (Basel). 2019 Aug 20;9(8):1182. doi: 10.3390/nano9081182 (PMC6724128; doi:10.3390/nano9081182)
Supplement: Supplementary file 1 [file nanomaterials-09-01182-s001.pdf]

nanomaterials

Supplementary material

Fabrication, Characterization, and Properties of Poly (Ethylene-Co-Vinyl Acetate) Composite Thin Films Doped with Piezoelectric Nanofillers

Giulia Mariotti and Lorenzo Vannozzi *

The BioRobotics Institute, Scuola Superiore Sant'Anna, Pontedera (PI) 56025, Italy
* Correspondence: lorenzo.vannozzi@santannapisa.it; Tel.: +39-050-883091

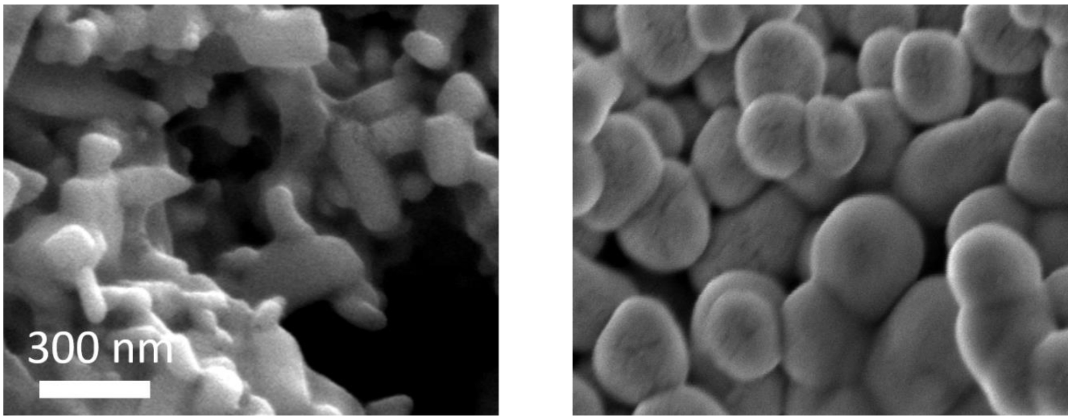

Figure S1. Dual Beam imaging of zinc oxide nanopowder (left) and barium titanate nanoparticles (right).

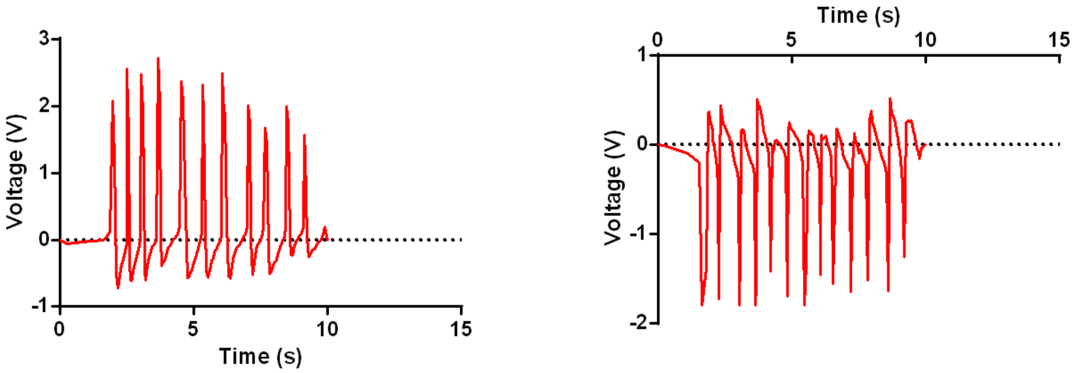

Figure S2. Voltage output in standard (left) and inverted (right) poles configuration.
